# Childhood socio-economic conditions and risk of cardiovascular disease: results from a pooled sample of 14 011 adults from India

**DOI:** 10.1136/jech-2020-214016

**Published:** 2020-10-01

**Authors:** Poppy Alice Carson Mallinson, Judith Lieber, Santhi Bhogadi, Sanjay Kinra

**Affiliations:** 1 Department of Non-Communicable Disease Epidemiology, London School of Hygiene and Tropical Medicine Faculty of Epidemiology and Population Health, London, UK; 2 Department of Population Health, London School of Hygiene and Tropical Medicine Faculty of Epidemiology and Population Health, London, UK; 3 Public Health Foundation of India, New Delhi, India

**Keywords:** CARDIOVASCULAR DISEASE, SOCIO-ECONOMIC, LIFECOURSE / CHILDHOOD CIRCUMSTANCES, OBESITY, PAEDIATRIC, NUTRITION, CHRONIC DI

## Abstract

**Background:**

South Asians are at an increased risk of premature cardiovascular disease, but the reasons for this are unclear. Poor socio-economic conditions in childhood are associated with an increased risk of cardiovascular disease in many high-income countries and may be particularly relevant to South Asia, where socio-economic deprivation is more prevalent and severe. However, evidence from South Asia is limited.

**Methods:**

We pooled data from two large population-based studies in India to provide a geographically representative and adequately powered sample of Indian adults. We used multilevel linear regression models to assess associations between standard of living index (SLI) in childhood (measured by recalled household assets at age 10–12 years) and major cardiovascular risk factors including adiposity, blood pressure, and fasting blood lipids, glucose and insulin.

**Results:**

Data on 14 011 adults (median age 39 years, 56% men) were analysed. SLI in childhood was inversely associated with systolic and diastolic blood pressure, independent of socio-economic conditions in adulthood, with beta coefficients (95% CIs) of −0.70 mmHg (−1.17 to −0.23) and −0.56 mmHg (−0.91 to −0.22), respectively, per SD increase in SLI in childhood. There was no strong evidence for an association between SLI in childhood and other risk factors of cardiovascular disease.

**Conclusions:**

Poor socio-economic conditions in childhood may contribute to the increased risk of premature cardiovascular disease among South Asians by raising their blood pressure. Elucidating the mechanisms and improving socio-economic conditions for children in South Asia could provide major reductions in the burden of cardiovascular disease.

## INTRODUCTION

Over a fifth of deaths from cardiovascular diseases globally occur in South Asia.^1^ Among South Asians, cardiovascular mortality is higher and occurs at younger ages than in other populations.^2 3^ Differences in behavioural risk factors in adulthood have failed to account for this observation.^4^ This has led some researchers to hypothesise a role for factors operating earlier in life.^5^

Evidence from high-income countries suggests that poor socio-economic conditions in childhood are associated with an increased risk of cardiovascular disease.^6 7^ Countries in South Asia are experiencing rapid economic development; until recently poor socio-economic conditions characterised by severe material deprivation were widespread, especially in rural areas.^8^ This suggests that the association of childhood socio-economic conditions with cardiovascular disease could be particularly strong in South Asia and account for the excess of premature cardiovascular diseases. Yet, the few studies from South Asia have failed to identify an association.^9 10^ Confirming this association and its pathways could inform the development of appropriate interventions for cardiovascular disease prevention in South Asia.

We examined the association of socio-economic conditions in childhood with a range of cardiovascular disease risk factors to investigate the potential pathways for an association. We pooled data from two population-based studies to provide a large sample representing different regions and varying levels of urbanisation in India.

## METHODS

We used data from two population-based studies: the Andhra Pradesh Children and Parents’ Study (APCAPS) and Indian Migration Study (IMS). Both studies were conducted by the same investigator teams using similar protocols, facilitating pooled analyses. APCAPS is a family-based cohort in 29 rural and peri-urban villages near Hyderabad in South India.^11^ It began as the long-term follow-up of offspring from a pregnancy nutrition trial conducted in 1987–1990 and was later expanded to cover family members of traced trial participants. In the present study, we used data from the third-wave survey conducted in 2010–2012, in which 6944 of 10 213 (68%) invited family members attended the clinical examination. The IMS was conducted in 2005–2007 to investigate the effects of rural to urban migration on the risk of cardiovascular disease.^12^ Factory workers and their spouses were recruited from four factory sites in urban centres (Lucknow, Nagpur, Hyderabad and Bangalore) and asked to invite their rural-dwelling siblings to the study. In total 7067 of 15 118 (47%) invited participants attended clinical examinations.

### Clinical assessment

Social and demographic information was collected using standard questions. These included a subset of questions from the standard of living index (SLI), which is an asset-based scale developed to measure household wealth in India.^13^ We only asked about items deemed most relevant for the study settings (11/29 questions on recalled household assets in childhood (age 10–12 years) and 14/29 questions on current household assets). These items were housing material, toilet facilities, lighting source, drinking water source and household possession of agricultural land, television, radio, clock, bicycle, motorcycle and refrigerator (plus household possession of a car, telephone and tractor for current assets). We derived the index by applying the recommended weights to the available household asset questions and then summing across all items to give a total score for each individual.^13^ A higher score indicates greater material affluence. Household-level measures of socio-economic conditions are particularly appropriate for use in India, where joint family structures render individual-level measures (such as occupation and education) less informative. We assessed socio-economic conditions in adulthood by SLI (as above) and by occupational status (categorised as unemployed/unskilled manual labour, skilled manual labour, non-manual/professional or student/housewife/retired), in order to capture multiple dimensions that might be relevant to the risk of cardiovascular disease.^14^

Anthropometric measurements (height, weight and waist circumference) were taken twice using standard equipment. Systolic blood pressure and diastolic blood pressure were measured at the right upper arm in the sitting position using a validated oscillometric device (Omron M5-I model). Participants were asked to rest for 5 minutes before taking multiple readings (three readings in APCAPS and two readings in IMS, each 1 minute apart).

Venous blood samples were drawn after a minimum of 8 hours fasting and centrifuged immediately. Assays for fasting glucose were conducted locally on the same day as sampling using enzymatic method. For all other assays, samples were transported in batches to our central laboratories for analysis. Total and high-density lipoprotein (HDL) cholesterol and triglycerides were estimated using enzymatic colorimetric methods and fasting insulin was estimated by radioimmunoassay. Quality of assays was assured by internal duplicates and regular external standards.

In both studies, we resurveyed a random 5% subsample of participants before the end of data collection to assess the reliability of the responses.

### Data analysis

We analysed data on the following cardiovascular risk factors: body mass index, waist circumference, systolic blood pressure, diastolic blood pressure and fasting total cholesterol, low-density lipoprotein (LDL) cholesterol, triglycerides, glucose, insulin and insulin resistance. We also examined the association of childhood socio-economic conditions with height because it is thought to be closely associated with childhood socio-economic conditions.^15^ Before analysis, we checked distributions of outcome variables and excluded extreme outliers. The mean of the first two blood pressure readings was used for consistency across studies. The mean of the two readings was used for all anthropometric measures. Body mass index was calculated as weight in kilograms/(height in metres)^2^. LDL cholesterol was estimated using the Friedewald–Fredrickson formula (Total cholesterol − HDL cholesterol − (triglycerides/5)).^16^ Homeostasis model assessment (HOMA) insulin resistance score was estimated as fasting insulin×fasting glucose/22.5.^17^ We applied a log transformation to the values of skewed variables (triglycerides, fasting glucose, insulin and HOMA score) to improve the normality of residuals.

To estimate the effect of SLI in childhood on cardiovascular risk factors in adulthood, we used multilevel linear regression models. We included a random intercept term at the family level to account for the potential correlation of cardiovascular risk factors between members of the same household and between sibling pairs. After first checking for evidence of non-linearity in its association with cardiovascular risk factors, we used SLI in childhood as a linear exposure. Age and sex are strongly associated with cardiovascular risk factors, so we included both as covariates in all models. We included linear, quadratic and cubic terms for age to account for its non-linear association with cardiovascular risk factors. We included a dummy variable for study (ie, APCAPS or IMS) to account for any systematic differences in measurement between the studies. Our primary models were further adjusted for place of residence (ie, urban or rural) and socio-economic conditions (measured by SLI and occupation) in adulthood. Socio-economic conditions in adulthood are highly correlated with socio-economic conditions in childhood and are directly associated with increased cardiovascular risk in India.^3 10 18^ Therefore, socio-economic conditions in adulthood could mask an inverse association between socio-economic conditions in childhood and risk of cardiovascular disease unless appropriately accounted for. We included a quadratic term for SLI in adulthood because there was some evidence of non-linearity in its association with cardiovascular disease risk factors. We checked for evidence of interactions between age and the other covariates (sex, study, place of residence and socio-economic conditions in adulthood) and included any substantial interactions in our final models to improve the precision of the estimates of interest. We repeated the analyses stratified by socio-economic conditions in adulthood (above or below median SLI) and by sex, as previous studies have suggested that these factors modify the association between childhood socio-economic conditions and cardiovascular risk factors.^19–21^ We also repeated the analyses stratified by study (ie, APCAPS or IMS) to investigate whether the associations of interest varied between the study populations.

To mitigate potential error in the recall of household assets in childhood, we fitted measurement error models using repeat data on a 5% subsample. We used the regression calibration method, which is an appropriate and efficient method when measurement error can be assumed to be non-differential with respect to the outcome.^22^ This assumption was reasonable in this case since levels of cardiovascular risk factors are generally not known to the participants and would therefore be unlikely to influence their recall of household assets in childhood. We regressed the repeat measure of the exposure on the original exposure (in the subsample), adjusting for covariates that may have influenced the reporting (ie, age, sex, adult socio-economic conditions). We used this model to predict a measurement error-adjusted exposure for all participants and used these predictions as the exposure variable in the main analyses. We estimated standard-errors using an adjusted delta method to account for the extra uncertainty from using predicted values as the exposure.^22^

We restricted all analyses to participants with complete exposure and outcome data, as a small number (<5%) were missing data for most outcomes, and participants with missing data were similar to those without. All analyses were performed using Stata 16.

### Ethical approval and role of the study sponsor

Ethical approval for the APCAPS and IMS studies was obtained from appropriate local review boards as well as the London School of Hygiene and Tropical Medicine. All participants provided written informed consent (or witnessed thumbprint if illiterate). The study sponsor had no role to play in the design, analysis, interpretation, write-up of this manuscript, or decision to submit for publication.

## RESULTS

**Figure 1:**
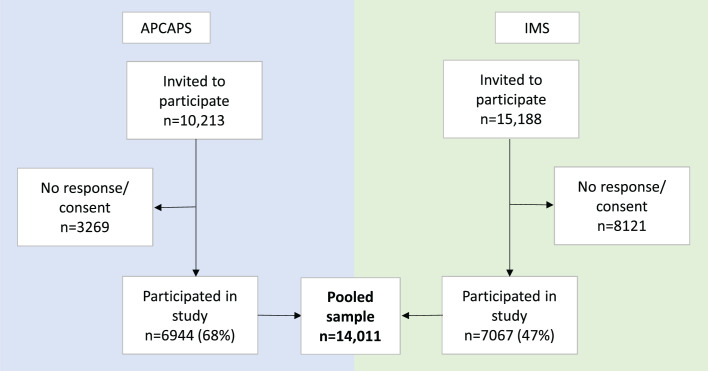
Flowchart of included study participants from the Indian Migration Study (IMS, 2005–2007) and Andhra Pradesh Children and Parents’ Study (APCAPS, 2010–2012).

We analysed data of 14 011 participants (6944 from APCAPS, 7067 from IMS, figure 1). The mean age of participants was 37.5 years and 56% were men (table 1). Complete data were available for 93%–99% of the participants, depending on the outcome. More outcome data were missing for APCAPS than IMS participants, although sociodemographic characteristics of participants with complete and incomplete outcome data were otherwise similar (supplementary material table S1).

**Table 1:** Description of study participants in pooled sample of IMS (2005–2007) and APCAPS (2010–2012)

			N (%)/mean (SD)		
Characteristics		% complete	APCAPS (N=6944)	IMS (N=7067)	Pooled sample (N=14 011)
*Sociodemographic*					
Age		>99%	34.2 (14.4)	40.8 (10.4)	37.5 (12.9)
Sex	Men	>99%	3646 (52.6%)	4123 (58.3%)	7769 (55.5%)
	Women		3283 (47.4%)	2944 (41.7%)	6226 (44.5%)
Childhood standard of living index		>99%	8.8 (4.8)	10.5 (5.1)	9.6 (5.0)
Adult standard of living index		>99%	18.6 (5.1)	23.3 (6.7)	21.0 (6.4)
Adult occupation	Unskilled labour or unemployed	>99%	2918 (42.1%)	314 (4.4%)	3232 (23.1%)
	Student, retired or housewife		2202 (31.8%)	2612 (37.0%)	4814 (34.4%)
	Semiskilled labour		732 (10.6%)	918 (13.0%)	1650 (11.8%)
	Skilled labour		709 (10.2%)	1548 (21.9%)	2257 (16.1%)
	Professional		365 (5.3%)	1675 (23.7%)	2040 (14.6%)
Adult residence	Rural	>99%	6944 (100%)	2668 (37.8%)	9612 (68.6%)
	Urban		0 (0%)	4399 (67.6%)	4399 (32.7%)
*Cardiovascular risk factors*					
Systolic blood pressure, mmHg		>99%	118.5 (15.3)	122.1 (17.0)	120.3 (16.3)
Diastolic blood pressure, mmHg		>99%	77.5 (12.7)	77.9 (11.0)	77.7 (11.9)
Total cholesterol, mmol/L		97%	4.2 (1.0)	4.7 (1.1)	4.5 (1.1)
LDL cholesterol, mmol/L		93%	2.8 (0.9)	3.3 (1.1)	3.1 (1.0)
Triglycerides, mmol/L (median, IQR)		95%	1.1 (0.8, 1.6)	1.3 (1.0, 1.8)	1.2 (0.9, 1.7)
Fasting glucose, mmol/L (median, IQR)		95%	5.0 (4.7, 5.4)	5.1 (4.6, 5.5)	5.1 (4.7, 5.5)
Insulin, mU/L (median, IQR)		95%	5.2 (3.1, 8.2)	6.0 (3.4, 9.6)	5.6 (3.2, 9.0)
HOMA score (median, IQR)		94%	1.2 (0.7, 1.9)	1.3 (0.8, 2.3)	1.3 (0.7, 2.1)
Body mass index, kg/m^2^		>99%	20.6 (3.9)	23.8 (4.5)	22.2 (4.5)
Waist circumference, cm		>99%	71.5 (10.7)	82.3 (11.7)	77.0 (12.5)
Height, cm		>99%	158.1 (9.5)	160.3 (8.8)	159.3 (9.3)

APCAPS, Andhra Pradesh Children and Parents’ Study; IMS, Indian Migration Study; LDL, low-density lipoprotein; HOMA, homeostasis model assessment.

10.1136/jech-2020-214016.supp1 Supplementary data

In age-adjusted and sex-adjusted analyses, SLI in childhood was directly associated with body mass index, waist circumference, total cholesterol, LDL cholesterol, triglycerides, glucose, insulin and HOMA score, but was not associated with blood pressure (table 2). After adjustment for socio-economic conditions in adulthood, SLI in childhood was inversely associated with systolic and diastolic blood pressure, with beta coefficients (95% CIs) of −0.70 mmHg (−1.17 to −0.23) and −0.56 mmHg (−0.91 to −0.22), respectively, per SD increase in childhood SLI. For all other cardiovascular risk factors, there was no strong evidence of an association with SLI in childhood. The direct association of height with SLI in childhood was hardly attenuated by adjustment for socio-economic conditions in adulthood, with adjusted beta coefficient (95% CI) of 5.1 mm (3.2–7.1) per SD increase in childhood SLI.

**Table 2:** Association between standard of living index (SLI) in childhood and cardiovascular risk factors in pooled sample of IMS (2005–2007) and APCAPS (2010–2012)

	Model 1: Age-adjusted and sex-adjusted					Model 2: model 1+ adult socioeconomic conditions*				
Cardiovascular risk factor	N	β-Coefficient for 1 SD change in childhood SLI	Lower confidence limit	Upper confidence limit	P-value	N	β-Coefficient for 1 SD change in childhood SLI	Lower confidence limit	Upper confidence limit	P-value
Systolic blood pressure, mmHg	13 931	−0.139	−0.562	0.284	0.520	13 931	−0.698	−1.165	−0.232	0.003
Diastolic blood pressure, mmHg	13 950	0.040	−0.275	0.355	0.805	13 950	−0.564	−0.912	−0.216	0.001
Total cholesterol, mmol/L	13 592	0.072	0.041	0.102	<0.001	13 592	0.006	−0.026	0.039	0.712
LDL cholesterol, mmol/L	12 974	0.055	0.027	0.083	<0.001	12 974	−0.010	−0.040	0.020	0.525
Log triglycerides, mmol/L	13 144	0.018	0.005	0.032	0.009	13 144	−0.009	−0.024	0.005	0.212
Log fasting glucose, mmol/L	13 224	0.015	0.009	0.020	<0.001	13 224	0.004	−0.002	0.009	0.174
Log insulin, mU/L	13 231	0.094	0.068	0.120	<0.001	13 231	0.021	−0.006	0.048	0.134
Log HOMA score	13 184	0.109	0.082	0.137	<0.001	13 184	0.025	−0.004	0.054	0.089
Body mass index, kg/m^2^	13 942	0.812	0.688	0.936	<0.001	13 942	0.083	−0.032	0.198	0.158
Waist circumference, mm	13 918	18.965	15.861	22.068	<0.001	13 918	−0.707	−3.629	2.216	0.636
Height, mm	13 942	9.076	7.168	10.984	<0.001	13 942	5.135	3.185	7.085	<0.001

APCAPS, Andhra Pradesh Children and Parents’ Study; HOMA, homeostasis model assessment; IMS, Indian Migration Study; LDL, low-density lipoprotein; SLI, standard of living index.

*Adult standard of living index (linear and quadratic term), adult occupation (categorical) and adult urban or rural residence (binary).

We found no strong evidence of effect modification by SLI in adulthood, sex or study for most of the cardiovascular risk factors (supplementary material tables S2, S3 and S4). An exception was for markers of adiposity: the direct association between SLI in childhood and waist circumference and body mass index was stronger among participants below the median SLI in adulthood than those above (p-interaction <0.001 and 0.01, respectively). Waist circumference was directly associated with SLI in childhood among men but inversely associated among women (p-interaction <0.001), while a reverse pattern was observed for body mass index (p-interaction=0.001). There was evidence of a direct association of childhood SLI with waist circumference and body mass index in IMS, but an inverse association in APCAPS (both p-interaction <0.001).

In analyses without accounting for measurement error in childhood SLI, results were similar but effect estimates were slightly attenuated (supplementary material table S5).

## DISCUSSION

### Main findings

In a large and geographically representative sample from India, we found that SLI in childhood was inversely associated with systolic and diastolic blood pressure in adulthood. There were no clear associations between SLI in childhood and other cardiovascular risk factors, including adiposity, total and LDL cholesterol, triglycerides, glucose, insulin and insulin resistance.

### Relation to literature

A large body of evidence from high-income countries supports an inverse association between socio-economic conditions in childhood and adiposity, blood pressure, cholesterol and insulin resistance, independent of socio-economic conditions in adulthood.^7 20 21 23–26^ Our findings, of an inverse association between childhood socio-economic conditions and blood pressure, but not with the other risk factors, suggest that different mechanisms are operating for different risk factors and that these are likely to be setting-specific. The lack of association we detected for the other risk factors, which are strongly linked to excess adiposity, may be due to relatively lower levels of adiposity in this study population. However, hardly any studies have been conducted in South Asia. A study of a birth cohort from Vellore in South India reported no evidence of association between parental education (an indicator of childhood socio-economic conditions) and overweight, hypertension, high triglycerides or diabetes at age 26–32 years.^10^ However, the CIs were wide and could have been consistent with either an inverse or a direct association. An earlier analysis of the IMS data alone (N=4120 men and N=2940 women) failed to detect an independent association of childhood SLI with systolic blood pressure, but this may have been due to inadequate adjustment for adult socio-economic conditions or lower statistical power (<30% power to detect the effect size found in our study as opposed to >99% power in our combined dataset).^9^ In our analyses, the inverse association of blood pressure with socio-economic conditions in childhood was masked until we adjusted for socio-economic conditions in adulthood, demonstrating the importance of adequate adjustment and large sample size to disentangle the effects of childhood from adult socio-economic conditions. In contrast, for the other risk factors, a crude direct association was attenuated to the null after adjustment for adult socio-economic conditions. This is consistent with the strong direct association between adult socio-economic conditions and adiposity observed in India (compared to the weak association for blood pressure).^27^

### Mechanisms

In this population, blood pressure was the only major cardiovascular risk factor associated with socio-economic conditions in childhood. Exposure to undernutrition during foetal development and early infancy has been linked to increased blood pressure in adulthood, for which a range of biological mechanisms have been proposed.^28^ However, evidence for this is inconclusive. In high-income countries, the link between early-life undernutrition and blood pressure has been largely inferred from studies of low birth weight, despite maternal undernutrition being rare and thus a less important cause of low birth weight. Subsequent observational studies and a community trial in South Asia have failed to confirm the association with blood pressure.^29–32^ Dehydration in infancy and early childhood caused by frequent diarrhoeal disease has been hypothesised to increase blood pressure in adulthood, although epidemiological evidence has been inconsistent.^33 34^ Emerging evidence suggests that frequent infection with malaria during childhood may also increase adult blood pressure, although confirmatory studies are needed.^35^ Other setting-specific environmental factors linked to poor socio-economic conditions, such as household use of biomass fuel, may play a role.^36^ However, evidence of the long-term effects of this and other putative local risk factors is lacking. Poor socio-economic conditions in childhood have also been linked to increased levels of risk behaviours (eg, smoking and alcohol consumption) and psychosocial adversity (eg, stress and depression) in adulthood, which are associated with increased blood pressure.^37^ However, the majority of evidence comes from high-income countries; further research is needed to understand the relevance of these mechanisms in South Asia.

### Strengths and limitations

Our study is one of the few studies from South Asia to have examined the association between childhood socio-economic conditions and cardiovascular risk factors. Our study had sufficient sample size to disentangle the effects of socio-economic conditions in childhood from adulthood, and statistical power was further improved by the use of regression calibration to reduce potential bias due to error in the recall of childhood assets used to derive the SLI. The study population represented different regions of India and varying levels of urbanisation making our results broadly generalisable to India. However, some limitations must be acknowledged. We cannot exclude the possibility of residual confounding by adult socio-economic conditions, which may have masked associations between childhood socio-economic conditions and other cardiovascular risk factors; however, this is unlikely to be the case given our use of multiple indicators for adult socio-economic conditions and sufficient sample size. There is also a possibility of residual bias due to differential recall of household assets in childhood. However, most participants were unaware of their cardiovascular risk factor levels, suggesting differential recall according to outcome status was unlikely. Furthermore, we noted a high degree of concordance in childhood SLI between siblings in the IMS (ρ=0.82) suggesting that participants were able to recall accurately. Previous studies have also noted accurate recall of childhood socio-economic circumstances and found this not to vary according to the educational status of the participants.^38 39^ Finally, we cannot exclude risk of selection bias because not all those invited chose to participate in the studies. However, analysis of baseline demographic characteristics of people who did and did not participate suggested few systematic differences.^11 12^

### Implications

Our findings suggest that poor socio-economic conditions in childhood could be contributing to the high cardiovascular disease burden in South Asia through their association with blood pressure. To put the effect sizes we observed into context, a study from the USA estimated that a 1 mmHg reduction in systolic or diastolic blood pressure across adults aged 45–64 could prevent approximately 30 events of coronary heart disease, stroke and heart failure per 100 000 person-years.^40^ If applied to the population of South Asia,^41^ this would equate to prevention of 185 000 premature cardiovascular events per year. Although there is a need for formal modelling of these estimates for South Asia, clearly the potential impact is appreciable. If these associations are proven to be causal, reductions in child poverty could have a meaningful impact on population blood pressure levels and associated cardiovascular events and could reduce social inequalities in cardiovascular disease. Further research should aim to confirm these findings in other South Asian settings and to elucidate the specific mechanisms underpinning the association, in order to inform setting-specific interventions for the primordial prevention of cardiovascular disease.

## CONCLUSION

We found that socio-economic conditions in childhood, independent of socio-economic conditions in adulthood, are inversely associated with blood pressure, but not associated with other cardiovascular risk factors. Improvements in childhood socio-economic conditions could have beneficial effects on adult blood pressure levels in South Asia. Further research is needed to understand the underlying mechanisms.

**What is already known on this subject**

- Poor socio-economic conditions in childhood are associated with increased risk of cardiovascular disease in many studies from high-income countries.
- This could be a potentially important contributor to the high mortality and premature occurrence of cardiovascular disease in South Asia, where socio-economic deprivation is both more prevalent and severe.
- However, there is hardly any evidence of this association from South Asian countries.

**What this study adds**

- In a large sample representing multiple geographic regions and levels of urbanicity within India, we found that poor socio-economic conditions in childhood were associated with increased systolic and diastolic blood pressure in adulthood.
- However, we did not find any association of socio-economic conditions in childhood with markers of adiposity, fasting blood lipids, glucose or insulin resistance, contrary to previous evidence.
- Childhood poverty, which remains highly prevalent in South Asia, may be contributing to the high burden of cardiovascular disease by increasing blood pressure. Elucidating the specific mechanisms involved could inform the design of appropriate interventions for prevention of cardiovascular diseases in South Asia.
