# Supplementary material for: Childhood socio-economic conditions and risk of cardiovascular disease: results from a pooled sample of 14 011 adults from India
Source: J Epidemiol Community Health. 2020 Oct 1;74(10):831–7. doi: 10.1136/jech-2020-214016 (PMC7577102; doi:10.1136/jech-2020-214016)
Supplement: Supplementary data [file jech-2020-214016s001.pdf]

Supplementary Material

Table S1: Differences between participants with complete and incomplete data on cardiovascular risk factors

Sociodemographic characteristics		N (%) / mean (standard deviation) Complete data (n=12,852)	Incomplete data (n=1111*)	p-value difference**
Study	APCAPS	6028 (46.9%)	868 (78.1%)	<0.001
	IMS	6824 (53.1%)	243 (21.9%)	
Age		37.6 (12.7)	36.6 (14.8)	0.02
Sex	Male	7144 (55.6%)	608 (54.7%)	0.55
	Female	5708 (44.4%)	503 (45.3%)	
Childhood household asset score		9.7 (5.0)	9.0 (5.1)	0.33
Adult household asset score		21.1 (6.5)	19.7 (6.0)	0.59
Adult occupation	Unskilled labour or unemployed	2884 (22.4%)	342 (30.8%)	0.08
	Student, retired or housewife	4399 (34.2%)	396 (35.6%)	
	Semi-skilled labour	1518 (11.8%)	131 (11.79%)	
	Skilled labour	2110 (16.4%)	146 (13.1%)	
	Professional	1941 (15.1%)	96 (8.6%)	
Adult residence	Rural	8622 (67.1%)	942 (84.8%)	0.02
	Urban	4230 (32.9%)	169 (15.2%)	

APCAPS=Andhra Pradesh Children and Parents' Study, IMS=Indian Migration Study
*Excluding 48 participants who did not have complete data on sociodemographic characteristics
**P-values based on likelihood ratio tests from logistic regression models adjusting for study (i.e. APCAPS or IMS)

Table S2: Association between standard of living index (SLI) in childhood and cardiovascular risk factors in pooled sample of IMS (2005-7) and APCAPS (2010-12) stratified by standard of living index in adulthood (above or below the median)

		Low standard of living index in adulthood (adjusted for age, sex and urban residence)			High standard of living index in adulthood (adjusted for age, sex and urban residence)			
Cardiovascular risk factor	N	β-coefficient for 1 SD change in childhood SLI	Lower confidence limit	Upper confidence limit	β-coefficient for 1 SD change in childhood SLI	Lower confidence limit	Upper confidence limit	P-value interaction
Systolic blood pressure, mmHg	13931	-0.209	-0.880	0.463	-0.323	-0.894	0.247	0.786
Diastolic blood pressure, mmHg	13950	-0.445	-0.947	0.058	-0.214	-0.640	0.211	0.466
Total cholesterol, mmol/L	13592	0.047	0.001	0.094	0.019	-0.021	0.058	0.324
LDL cholesterol, mmol/L	12974	0.033	-0.011	0.076	0.004	-0.033	0.040	0.281
Log triglycerides, mmol/L	13144	0.006	-0.016	0.028	-0.006	-0.025	0.012	0.381
Log fasting glucose, mmol/L	13224	0.011	0.003	0.019	0.005	-0.002	0.011	0.208
Log insulin, mU/L	13231	0.063	0.023	0.103	0.039	0.005	0.072	0.325
Log HOMA score	13184	0.072	0.030	0.114	0.046	0.010	0.081	0.306
Body mass index, kg/m^2	13942	0.430	0.262	0.599	0.161	0.019	0.304	0.010
Waist circumference, mm	13918	13.80	9.431	18.18	1.623	-2.001	5.246	<0.001
Height, mm	13942	7.094	4.300	9.888	7.308	4.916	9.701	0.900

APCAPS=Andhra Pradesh Children and Parents' Study, IMS=Indian Migration Study, SD=standard deviation, SLI=standard of living index, LDL=low-density lipoprotein, HOMA=homeostasis model assessment

Table S3: Association between standard of living index (SLI) in childhood and cardiovascular risk factors in pooled sample of IMS (2005-7) and APCAPS (2010-12), stratified by sex.

		Females (adjusted for age and adult socioeconomic conditions*)			Males (adjusted for age and adult socioeconomic conditions*)			
Cardiovascular risk factor	N	β-coefficient for 1 SD change in childhood SLI	Lower confidence limit	Upper confidence limit	β-coefficient for 1 SD change in childhood SLI	Lower confidence limit	Upper confidence limit	P-value interaction
Systolic blood pressure, mmHg	13931	-0.851	-1.489	-0.213	-0.350	-0.951	0.252	0.193
Diastolic blood pressure, mmHg	13950	-0.558	-1.034	-0.083	-0.592	-1.043	-0.142	0.906
Total cholesterol, mmol/L	13592	-0.004	-0.048	0.040	0.009	-0.033	0.050	0.625
LDL cholesterol, mmol/L	12974	-0.024	-0.065	0.017	0.000	-0.039	0.039	0.321
Log triglycerides, mmol/L	13144	-0.008	-0.028	0.013	-0.015	-0.035	0.004	0.554
Log fasting glucose, mmol/L	13224	-0.001	-0.008	0.007	0.004	-0.003	0.011	0.289
Log insulin, mU/L	13231	-0.021	-0.058	0.016	0.043	0.008	0.078	0.005
Log HOMA score	13184	-0.020	-0.059	0.019	0.046	0.009	0.083	0.006
Body mass index, kg/m^2	13942	0.206	0.049	0.363	-0.110	-0.259	0.039	0.001
Waist circumference, mm	13918	-6.709	-10.72	-2.697	4.838	1.055	8.621	<0.001
Height, mm	13942	5.523	2.882	8.164	5.396	2.904	7.887	0.935

APCAPS=Andhra Pradesh Children and Parents' Study, IMS=Indian Migration Study, SD=standard deviation, SLI=standard of living index, LDL=low-density lipoprotein, HOMA=homeostasis model assessment

*Adult standard of living index (linear and quadratic term), adult occupation (categorical) and adult urban or rural residence (binary)

Table S4: Association between standard of living index (SLI) in childhood and cardiovascular risk factors in pooled sample of IMS (2005-7) and APCAPS (2010-12), stratified by study (APCAPS or IMS).

		APCAPS (adjusted for age, sex and adult socioeconomic conditions*)			IMS (adjusted for age, sex and adult socioeconomic conditions*)			
Cardiovascular risk factor	N	β-coefficient for 1 SD change in childhood SLI	Lower confidence limit	Upper confidence limit	β-coefficient for 1 SD change in childhood SLI	Lower confidence limit	Upper confidence limit	P-value interaction
Systolic blood pressure, mmHg	13931	-0.199	-0.961	0.564	-0.938	-1.718	-0.158	0.224
Diastolic blood pressure, mmHg	13950	-0.294	-0.864	0.275	-0.853	-1.434	-0.272	0.216
Total cholesterol, mmol/L	13592	-0.035	-0.088	0.017	0.039	-0.015	0.094	0.077
LDL cholesterol, mmol/L	12974	-0.030	-0.079	0.020	0.006	-0.043	0.055	0.364
Log triglycerides, mmol/L	13144	-0.028	-0.054	-0.003	0.003	-0.021	0.027	0.108
Log fasting glucose, mmol/L	13224	-0.010	-0.019	-0.001	0.012	0.003	0.021	0.002
Log insulin, mU/L	13231	-0.010	-0.055	0.036	0.037	-0.008	0.081	0.190
Log HOMA score	13184	-0.018	-0.066	0.029	0.048	0.001	0.095	0.078
Body mass index, kg/m^2	13942	-0.317	-0.506	-0.128	0.368	0.173	0.564	<0.001
Waist circumference, mm	13918	-9.505	-14.34	-4.673	8.682	3.747	13.616	<0.001
Height, mm	13942	6.328	3.204	9.452	4.599	1.297	7.900	0.494

APCAPS=Andhra Pradesh Children and Parents' Study, IMS=Indian Migration Study, SD=standard deviation, SLI=standard of living index, LDL=low-density lipoprotein, HOMA=homeostasis model assessment

*Adult standard of living index (linear and quadratic term), adult occupation (categorical) and adult urban or rural residence (binary)

Table S5: Association between standard of living index (SLI) in childhood and cardiovascular risk factors in pooled sample of IMS (2005-7) and APCAPS (2010-12), not accounting for measurement error in childhood standard of living index.

	Model 1: Age- and sex-adjusted					Model 2: model 1 + adult socioeconomic conditions*				
Cardiovascular risk factor	N	β-coefficient for 1 SD change in childhood SLI	Lower confidence limit	Upper confidence limit	p-value	N	β-coefficient for 1 SD change in childhood SLI	Lower confidence limit	Upper confidence limit	p-value
Systolic blood pressure, mmHg	13943	-0.169	-0.456	0.118	0.248	13931	-0.512	-0.826	-0.198	0.001
Diastolic blood pressure, mmHg	13962	-0.049	-0.263	0.165	0.653	13950	-0.444	-0.678	-0.210	<0.001
Total cholesterol, mmol/L	13604	0.041	0.021	0.062	<0.001	13592	-0.001	-0.023	0.021	0.947
LDL cholesterol, mmol/L	12984	0.029	0.010	0.048	0.003	12974	-0.013	-0.033	0.008	0.217
Log triglycerides, mmol/L	13154	0.011	0.002	0.020	0.021	13144	-0.007	-0.018	0.003	0.146
Log fasting glucose, mmol/L	13235	0.009	0.006	0.012	<0.001	13224	0.002	-0.001	0.006	0.196
Log insulin, mU/L	13242	0.060	0.042	0.077	<0.001	13231	0.011	-0.008	0.029	0.266
Log HOMA score	13195	0.069	0.051	0.087	<0.001	13184	0.013	-0.006	0.033	0.183
Body mass index, kg/m^2	13954	0.515	0.440	0.590	<0.001	13942	0.045	-0.033	0.123	0.258
Waist circumference, mm	13930	12.48	10.59	14.37	<0.001	13918	-0.034	-2.017	1.948	0.973
Height, mm	13954	5.530	4.303	6.758	<0.001	13942	3.060	1.757	4.364	<0.001

APCAPS=Andhra Pradesh Children and Parents' Study, IMS=Indian Migration Study, SD=standard deviation, SLI=standard of living index, LDL=low-density lipoprotein, HOMA=homeostasis model assessment

*Adult standard of living index (linear and quadratic terms), adult occupation (categorical) and adult urban or rural residence (binary)
